# Transporting to the matrix: A pyrenoid-localized phosphate transporter required for optimal photoautotrophic growth in algae

**DOI:** 10.1093/plphys/kiaf232

**Published:** 2025-06-06

**Authors:** James M Bradley

**Affiliations:** Assistant Features Editor, Plant Physiology, American Society of Plant Biologists; Department of Cell & Systems Biology, University of Toronto, Toronto, ON, Canada M5S 3B2

Approximately 50% of total global carbon fixation is achieved through photosynthesis by algae (Field et al. 1998). Perhaps the best studied algal species is the model *Chlamydomonas reinhardtii*, affectionately called “Chlamy” (Salomé and Merchant 2019; Dupuis and Merchant 2023). Like all algae, Chlamy uses the enzyme Rubisco to capture CO_2_ during photosynthesis. However, Rubisco is notoriously inefficient and in addition to its useful carboxylation reaction can also catalyze an energetically wasteful oxygenation reaction, particularly when CO_2_ is limiting. The algal solution to this problem was to evolve a carbon concentrating mechanism (CCM) that concentrates CO_2_ around Rubisco and involves a specialized compartment within the chloroplast called the “pyrenoid” (He et al. 2023). The pyrenoid consists of a matrix of Rubisco and other proteins that form a biomolecular condensate surrounded by a starch sheath and traversed by thylakoid tubules (Figure A). To concentrate CO_2_ within the matrix, bicarbonate ions (${\mathrm{HCO}}_{3}^{-}$) are first pumped into the thylakoid lumen, which then diffuse through the connected thylakoid tubules. There, ${\mathrm{HCO}}_{3}^{-}$ ions are converted by carbonic anhydrase back to CO_2_, which can then diffuse into the pyrenoid matrix for capture by Rubisco (Figure A) (He et al. 2023; Catherall et al. 2025 ). The products of Rubisco CO_2_ fixation are then moved into the chloroplast stroma to participate in the remaining steps of the Calvin-Benson cycle that generates organic molecules required for life.

**Figure.**
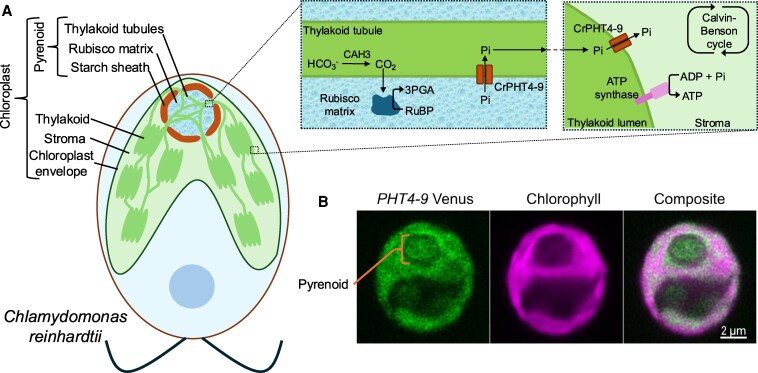
CrPHT4-9 is a thylakoid and pyrenoid localized Pi transporter in *Chlamydomonas reinhardtii*. **A)** A *Chlamydomonas reinhardtii* (“Chlamy”) cell indicating the chloroplast and pyrenoid. The pyrenoid is an ephemeral biomolecular condensate consisting of Rubisco in a matrix of other proteins. The pyrenoid concentrates CO_2_ around Rubisco to permit efficient production of 3-phosphoglycerate (3PGA) from ribulose-1,5-bisphosphate (RuBP). Traversing the pyrenoid matrix are thylakoid tubules that bring bicarbonate ions (${\mathrm{HCO}}_{3}^{-}$) into the pyrenoid. Carbonic anhydrase 3 (CAH3) converts ${\mathrm{HCO}}_{3}^{-}$ to CO_2_, which can then diffuse into the Rubisco matrix (He et al. 2023). The remaining steps of the Calvin-Benson cycle occur in the chloroplast stroma (Küken et al. 2018). To facilitate the spatially separated biochemistry, Pi (and other molecules) must move between the pyrenoid matrix and the stroma. Shaikh et al. found CrPHT4-9 is a thylakoid membrane-localized Pi transporter likely involved in transporting Pi across the thylakoid membranes to permit optimal photoautotrophic growth. **B)** Confocal images for wild-type Chlamy expressing a CrPHT4-9-Venus reporter. The protein is localized to the chloroplast thylakoid membrane and thylakoid tubules that traverse the pyrenoid matrix. Images reproduced from Shaikh et al. (2025).

A major research goal is to engineer pyrenoid-like subcellular compartments into land plant chloroplasts (He et al. 2023). This is predicted to supercharge carbon fixation and could have profound impacts on agricultural productivity. Although progress has been made—a proto-pyrenoid was recently reconstituted in *Arabidopsis* chloroplasts (Atkinson et al. 2020)—a fully functional pyrenoid has not yet been established in plant chloroplasts (Catherall et al. 2025). To achieve this, it will be important to understand how ions and metabolites move efficiently between the chloroplast stroma and the pyrenoid matrix via the thylakoid tubules (Atkinson et al. 2020). For example, inorganic phosphate (Pi) is vital for cellular biochemistry and ATP synthesis, and thus the flux of Pi between the stroma and the pyrenoid matrix is likely to be important for a fully functional CCM in Chlamy. Indeed, the pyrenoid matrix hosts a highly active metabolism that includes phosphorylation and dephosphorylation reactions (Zhan et al. 2018; He et al. 2023). However, the molecular mechanism by which Pi is transported across the thylakoid membranes remains unknown.

Recently in *Plant Physiology*, Shaikh et al. (2025) set out to identify transporters in Chlamy involved in Pi flux across the thylakoid membranes. Since the pyrenoid is important for optimal photoautotrophic growth under limiting CO_2_, they searched for putative transporter mutants that had growth defects under these conditions (based on high-throughput screens conducted by Fauser et al. 2022). They landed on a putative Pi transporter, CrPHT4-9. Comparing the amino acid sequence with Pi transporters from land plants, algae, diatoms, and bacteria, they found CrPHT4-9 most closely aligned with the PHT4 family from land plants and green algae. Most members of this gene family are predicted to localize to the chloroplast (Wang et al. 2020). Indeed, a recent study showed another PHT4 family member, CrPHT4-7, localized to the chloroplast outer envelope and was important for Pi transport into the chloroplast stroma (Tóth et al. 2024). To investigate the precise subcellular localization pattern of CrPHT4-9, the authors made a CrPHT4-9-Venus fusion and observed localization *within* the chloroplast (Figure B), rather than the outer envelope as seen for CrPHT4-7 (Tóth et al. 2024). Crucially, the fluorescent signal extended into the pyrenoid matrix, presumably along the thylakoid tubules (Figure B). This strongly suggested CrPHT4-9 is embedded within the thylakoid membranes and might be moving Pi between the pyrenoid matrix and the thylakoid lumen and/or chloroplast stroma (Figure B).

To test the ability of CrPHT4-9 to transport Pi, Shaikh et al. expressed CrPHT4-9 in *Saccharomyces cerevisiae* (yeast) cells and measured growth as a proxy for Pi uptake. To eliminate the yeast's own Pi transport function, they used a special strain called EY917, which was engineered to lack all native Pi transporters (Wykoff and O’Shea 2001). How then does EY917 survive if it cannot uptake Pi? The trick was to introduce a plasmid-borne gene encoding the yeast high-affinity Pi transporter, *ScPHO84*, under the control of the galactose-inducible *GAL1* promoter (Wykoff and O’Shea 2001). Thus, EY917 growth is permissible when galactose is present as the sole carbon source, as *ScPHO84* is expressed to allow Pi uptake. When glucose is added, expression from the *GAL1* promoter is strongly repressed, meaning *ScPHO84* is no longer transcribed and Pi uptake and growth are abolished. Shaikh et al. introduced a constitutively expressed copy of CrPHT4-9 on a second plasmid into EY917 and observed growth in glucose, indicating that CrPHT4-9 is competent to transport Pi in yeast. The authors found their strain grew slower than the wild type, but this could be explained if CrPHT4-9 is not efficiently targeted to the yeast plasma membrane, which is not unexpected for an algal protein.

The observation that CrPHT4-9 was localized to the pyrenoid suggested a potential role in the CCM. Since the CCM is vital for optimal growth under photosynthetic conditions, the authors also predicted CrPHT4-9 might be important specifically for photoautotrophic growth in Chlamy. Importantly, Chlamy is a facultative autotroph, meaning it can either conduct photoautotrophic growth by fixing CO_2_ through photosynthesis or undergo nonphotoautotrophic growth by using a reduced carbon source, such as acetate (Salomé and Merchant 2019). For example, under dark conditions in the presence of acetate, Chlamy can grow well without relying on the pyrenoid for CO_2_ fixation. This property of Chlamy allowed Shaikh et al. to test the role that CrPHT4-9 plays in photoautotrophic vs nonphotoautotrophic growth. They acquired 2 independent mutant lines (*pht4-9.1* and *pht4-9.2*) and assessed their growth in light and dark/acetate conditions. As predicted, growth of both *pht4-9* mutant alleles was inhibited only in the light and rescued when complimented with a wild-type gene.

Given the importance of CrPHT4-9 to photoautotrophic growth of Chlamy, the authors next assessed the photosynthetic performance of their *pht4-9* mutants. When chlorophyll is excited by light, the energy can drive photosynthesis or be re-emitted as either heat (nonphotochemical quenching) or chlorophyll fluorescence (Murchie and Lawson 2013). Because these 3 processes occur in competition, measuring one provides information on the other (Murchie and Lawson 2013). Thus, by measuring chlorophyll fluorescence, Shaikh et al. could show that the *pht4-9* mutants had significantly lower photosynthetic performance overall compared with wild type. This was accompanied by reduced electron transport rates and increased nonphotochemical quenching. Since the electron transport chain supports the production of ATP, the authors also measured the activity of ATP synthase. Using electrochromic shift assays, they showed *pht4-9* mutants had reduced proton flux across the thylakoid membranes; this was attributed to reduced ATP synthase activity. Finally, Shaikh et al. tested the prediction that their *pht4-9* mutants were compromised in the CCM. To do this, they fed their cells 2% CO_2_, which is considered high compared with ambient levels of 0.03% to 0.05% (Catherall et al. 2025). Under 2% CO_2_, the CCM becomes much less important for optimal photoautotrophic growth, and accordingly Shaikh et al. found their treatment rescued the mutant growth defect previously observed. Putting their data together, the authors proposed that CrPHT4-9 is required to move Pi from the pyrenoid matrix to the chloroplast stroma, which indirectly supports the CCM activity and promotes ATP synthesis (Figure A).

In sum, Shaikh et al. have characterized a thylakoid- and pyrenoid-localized Pi transporter from the model green algae, *Chlamydomonas reinhardtii*. Their study highlights the importance of correct Pi flux between the pyrenoid matrix and the stroma for optimal photoautotrophic growth and correct functioning of the CCM within the pyrenoid matrix. Understanding the full complement and functioning of transporters required for the CCM could help engineer a pyrenoid-like compartment into land plant chloroplasts, which is predicted to dramatically increase photosynthetic efficiency (Catherall et al. 2025).

## Data Availability

No new data were generated or analyzed in support of this research.
